# Detecting Cancer Outlier Genes with Potential Rearrangement Using Gene Expression Data and Biological Networks

**DOI:** 10.1155/2012/373506

**Published:** 2012-06-28

**Authors:** Mohammed Alshalalfa, Tarek A. Bismar, Reda Alhajj

**Affiliations:** ^1^Department of Computer Science, University of Calgary, Calgary, AB, Canada T2N 1N4; ^2^Departments of Pathology, Oncology and Molecular Biology and Biochemistry, Faculty of Medicine, University of Calgary, Calgary, AB, Canada T2N 1N4

## Abstract

Gene alterations are a major component of the landscape of tumor genomes. To assess the significance of these alterations in the development of prostate cancer, it is necessary to identify these alterations and analyze them from systems biology perspective. Here, we present a new method (EigFusion) for predicting outlier genes with potential gene rearrangement. EigFusion demonstrated excellent performance in identifying outlier genes with potential rearrangement by testing it to synthetic and real data to evaluate performance. EigFusion was able to identify previously unrecognized genes such as *FABP5 * and * KCNH8* and confirmed their association with primary and metastatic prostate samples while confirmed the metastatic specificity for other genes such as *PAH, TOP2A,* and *SPINK1*. We performed protein network based approaches to analyze the network context of potential rearranged genes. Functional gene rearrangement Modules are constructed by integrating functional protein networks. Rearranged genes showed to be highly connected to well-known altered genes in cancer such as *AR, RB1, MYC,* and *BRCA1*. Finally, using clinical outcome data of prostate cancer patients, potential rearranged genes demonstrated significant association with prostate cancer specific death.

## 1. Introduction

**Figure 1:**
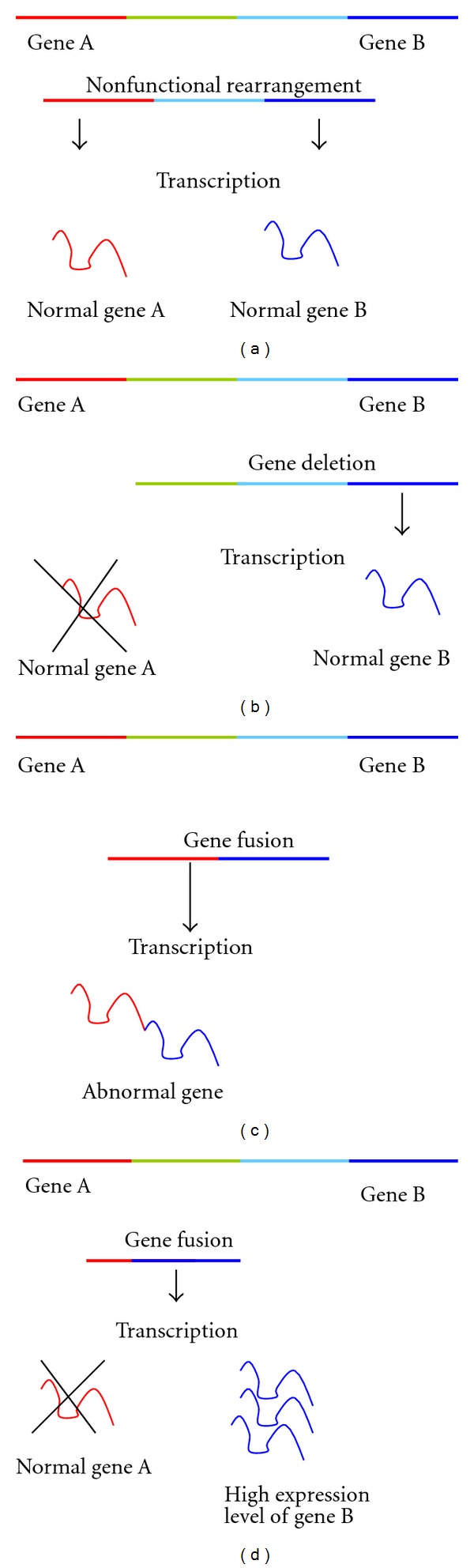
Gene rearrangements, common gene rearrangements in cancer cells. (b) Shows the deletion of some genes at the DNA level which leads to depletion of corresponding mRNA. (c) Represents the fusion of two genes that leads to fused mRNA and fused proteins. (d) Represents special rearrangement type that fuses a strong promoter of gene A to the 5' of gene B. This leads to underexpression of gene A and overexpression of gene B.

Genetic alterations in cancer are the most challenging factors that might lead to aggressive behavior of cells. Among the most prevalent forms of genetic alterations observed in cancer cells are gene fusions, gene amplification, and gene deletions. Recurrent translocations generally fall into two categories: functional rearrangements that result in a change in gene's activity due either to a change in protein quality or quantity and the other category is silent translocations that have no effect on gene's activity. Functional translocations can be categorized into two subtypes; one that leads to fused transcripts resulting in new proteins with different activity like * BCR-ABL* in leukemia [1] and * EML4-ALK* in lung cancer [2]; on the other hand, it can lead to change in a transcript quantity by translocating a strong gene promoter to the intact coding region of an oncogene like * TMPRSS2-ERG* [3]. Another functional genomics rearrangement is genomic deletion which results in loss of DNA segment that might harbour functional genes. * PTEN *is a well-studied genomic deletion in prostate cancer that is anticipated to trigger a cascade of genomic rearrangements [4].  Figure 1 gives a schematic description of the four rearrangement types.

Identifying gene rearrangements in general and gene amplification and deletions in particular has been a challenge during the past decade as it requires deep DNA sequence analysis of many cancer samples and their paired counterparts [5]. Though sequencing cancer genomes can reveal very precise results about gene fusion or deletions, it is not an easy task to obtain sequence data, as this needs fresh tissue and still relatively expensive. Another method to detect gene rearrangements, namely gene fusion, is to design special oligo microarray which covers all possible genomic rearrangements [6]. This method requires some knowledge about the predicted gene fusion variants and all possible exon-exon junctions. Recently, RNA-Sequencing have gained attention to identify novel gene fusions [7]. Sequence reads that align across a gene fusion boundary (so-called split reads) are a strong source of evidence for gene fusions in paired-end RNA-Seq data. A number of algorithms and tools have been proposed to find split reads such as PERAlign [8], MapSplice [9], and deFUSION [10]. The only advantage of RNA-Seq-based algorithms is that they are able to discover fused transcripts (Figure 1(c)), but they are unable to discover rearranged genes at the DNA level (Figure 1(d)) as these types of rearrangements are not reflected in the RNA sequence. Despite the high accuracy of the above-described data types to discover gene rearrangements, obtaining and analyzing (RNA-Seq, oligo-microarray, deep sequencing) data is very expensive and extremely challenging as it depends on sequence assembly and alignment algorithms.

Another source of information to identify potential functional gene rearrangements or gene alterations that affect gene expression is microarray gene expression data that we will use in this work to discover gene rearrangements. Unlike sequence data that can be easily interpreted to identify gene rearrangements, microarray gene expression data requires preprocessing steps. New direction to detect genomic rearrangements is to use bioinformatics approaches applied to gene expression data [11]. This problem is different from the detection of biomarker genes in several aspects. Biomarkers are differentially expressed in almost all samples, while gene rearrangements occur in only a subset of samples. Many studies showed that common biomarker extraction methods such as *t*-test, SAM, *and so forth* are not proper for detecting gene rearrangements [12, 13] as these studies attempt to maximize the difference between all cancer samples against all normal samples. Since fusion genes and gene deletions are rare genomic events leading to over expression in subset of cancer samples, new specialized computational approaches are in need to solve this problem.

Several methods have been proposed to identify rearranged genes from gene expression data (methods are described in supplementary file available on line at doi:10.1155/2012/373506). In the context of this work, we use outlier genes to refer to potential rearranged genes or altered genes. All previous methods consider each gene individually when ranking genes. However, ranking based on the global properties of the genes would reduce error rates. Herein, we show that all existing methods detect biomarker genes as outlier genes; a drawback of existing methods that we solved in our approach by proposing a new transformation function. The second advantage of the proposed method is simultaneous detection of potential gene amplification and potential gene deletions. None of the previous methods were reported to detect gene deletions, though they can be modified slightly to achieve the task of gene deletion detection. Thus, proposing different methods that can assess the over expression of a subset of genes is highly desirable for detecting gene rearrangements using microarray gene expression data.

In this paper, we use cancer cohorts (microarray gene expression data of hundreds of samples) to identify outlier genes that are overexpressed or underexpressed in subset of cancer samples using gene expression data. Outlier genes with overexpression are anticipated to have potential gene rearrangements, and outlier genes with underexpression are anticipated to have potential deletion. We propose EigFusion method that ranks outlier genes based on their effect on the gene expression matrix largest eigenvalue when removed from data; this effect could be due to gene overexpression in subset of samples or underexpression in subset of samples. After identifying outlier genes with potential rearrangement, outlier genes are characterized from a systems biology angle. Network is constructed to link potential rearranged genes by integrating functional protein networks to identify modules enriched with potential rearranged genes. Finally, we assess the clinical significance of the predicted rearrangements using clinical and survival data.

## 2. Materials and Methods 

### 2.1. Existing Statistical Methods

Here we define annotations for the gene expression data, genes, and samples that we will use across this paper in both the existing methods and the proposed method section. Let *X*_*ij*_ be the expression values for genes *i* = 1,2, ..., *m* and samples *j* = 1,2, ..., *n*. We assume that samples are grouped into two groups *S*1 and *S*2. In our work, *S*1 represents cancer samples and *S*2 represents normal samples.

In this work, we used all existing methods that are designed to tackle this computational problem. Cancer Outlier Profile Analysis (COPA) [14] is considered as the first algorithm that lead to the discovery of * ERG* rearrangement in prostate cancer. Outlier sums [15] were introduced to improve the *r*th percentile factor of COPA. The outlier robust *t*-statistic [12] is very similar to OS but it replaces the overall median by the median of normal samples. Another algorithm is the GTI algorithm [16] that weights the proportion of outliers by a robust measure of how outlying the outliers are in a single group. The previous four methods test for genes that are overexpressed in a subset of cancer samples regardless of the expression value of the remaining subset from cancer samples. This might lead to false positives as the remaining subset in cancer samples should be normally expressed. The methods are described in details in the supplementary file. Thus, there is a need for a more robust method that is not single genes based, is able to discriminate between biomarkers and outlier genes, and is not sensitive to the portions of cancer samples in the dataset. As a consequence, we propose EigFusion as an effective method to identify outlier genes with potential gene rearrangements from microarray gene expression data.

### 2.2. The Proposed EigFusion Method

A new method called EigFusion is proposed to predict genes that are overexpressed (potential fusion genes or amplified) or underexpressed (potential deletion genes) in subset of samples using gene expression data. EigFusion standardizes the gene profile based on a newly defined median value for cancer samples. One of the important factors to determine in standardizing the profiles is to decide on the median. COPA and OS use the overall median, but ORT uses the median of normal samples. We think the median might be very crucial to distinguish between outlier genes and biomarkers. Thus, in here, we use the median of cancer samples to standardize the gene expression values across all samples. As a result, genes with high expression values in all cancer samples can be filtered out. Since some rearrangements might be more frequent for some genes and might occur in more than half of the cancer samples, we define three median values for each gene. The first one is the median of cancer samples (*median* ^*S*1^), we then divide cancer samples into two groups: values greater than *median* ^*S*1^ and values less than *median* ^*S*1^. We used the average of the medians of the three groups, we call it *A* *V* *G* *median* ^*S*1^. We defined the transformation function as

(1)X^ij=Xij−AVGmedianiS1median(|xij−mediani|),

where *median*(*i*) is the median of *gene*(*i*) whole profile.

After transforming the expression values, genes were ranked using the following formula:

(2)Scorei=E(X^i)∗(E(X^iS1)−E(X^iS2))E(X^m×ni),

where ${\hat{X}}_{i}$ is the transformed expression profile of *gene*(*i*) across all samples and $E({\hat{X}}_{i})$ is the largest eigenvalue of transformed *gene*(*i*) after converting it to matrix by multiplying it by its transpose, $E({\hat{x}}_{i}^{S1})$ is the largest eigenvalue of *gene*(*i*) in cancer samples, $E({\hat{x}}_{i}^{S2})$ is the largest eigenvalue of *gene*(*i*) in normal samples, and $E({\hat{X}}_{m\times n}^{i})$ is the largest eigenvalue of the matrix that have all the genes across all samples without *gene*(*i*). The eigenvalues are large when the expression values are high; thus, when genes have high expression values in subset of cancer samples, they will be ranked high.

### 2.3. Gene Expression Data Simulation

 Synthetic gene expression data was generated from a standard normal distribution *N*(*μ*, *δ* ^2^). Gaussian noise *ϵ* was added to the expression values. Expression values of 1000 genes across 200 samples were simulated, and 10 test genes were added to evaluate the performance of the algorithms. Test genes were generated by adding a constant *u* (the maximum value in the data) to the expression of *k* cancer samples in the test genes, where *k* is chosen to be 2, 5, 10, 20, 50, 80, 100, 120, or 150; test genes are represented as *test*_*k*_. For example, *test*_10_ means that there are 10 cancer samples with added constant *u*; this gene represents an outlier gene that is amplified in 10 cancer samples. We also divided the 200 samples into cancer and normal groups. We used different sizes for the cancer group; we used size 20, 50, 100, 120, 150, or 180 samples. The aim from this variation in the size of the cancer samples is to evaluate the performance of the algorithms at different ratios of cancer to normal samples and assess the statistical power of EigFusion algorithm. In addition, test genes and size variation are critical to show the drawbacks of existing methods and how EigFusion can overcome the drawbacks of the existing methods.

### 2.4. Protein Module Rearrangements Enrichment

 We next integrated functional protein networks to assess if rearranged genes are functionally related and form modules. Functional gene rearrangements are anticipated to have effect on proteins associated with them. We integrated the identified potential rearranged genes with a functional protein interaction (FPI) network that covers more than half of the human genes and has more than 180,000 interactions. FPI was constructed from several data sources (Reactome, KEGG, CellMap, human PPI) as described in [17]. Modules with enriched rearranged genes were further characterized. Reactome FI cytoscape plugin was used to visualize and cluster the rearranged genes network [17].

## 3. Results

### 3.1. EigFusion Performance Evaluation on Simulated Data

The receiver operating characteristics (ROC) curve was used for evaluating the performance of the different statistical methods and compare them with the proposed method. ROC curves were constructed using sensitivity and specificity rates for each method under each cancer samples' size; a variable that we used to assess the performance of our method to distinguish between biomarkers and potential rearranged genes. ROC curves showed to be not very sensitive to false discoveries. Therefore, we used three statistical measures to assess the false discovery rate of the methods. We used false positive discovery rate (FPR)(FP/FP + TP), false negative discovery rate (FNR)(FN/FN + TN), and f-measure defined as

(3)f−measure=2∗precision∗recallprecision+recall,

where precision is (TP/TP + FP), and recall is (TP/TP + FN).

FP is detected when the method ranks a gene in the top 10 when it is supposed to be ranked very low in the ranking list. For example, when the cancer sample size is 50 or 100, most algorithms (excluding EigFusion) ranked *test*_50_ and *test*_100_ genes, respectively, at the top of the list. We consider this as a false positive because these genes are supposed to be biomarkers as they have high expression in all cancer samples. None of the other methods were able to distinguish between biomarker genes and rearranged genes because they standardize expression profile with respect to overall median. When normal sample size is greater than cancer sample size, the median will be biased toward normal samples. Therefore, biomarker genes will not be filtered out as they will satisfy the IQR threshold and they will be ranked high. FN is detected when the method ranks positive test genes, which have high expression in less than half of the cancer samples, at the bottom of the ranking list.

**Figure 2:**
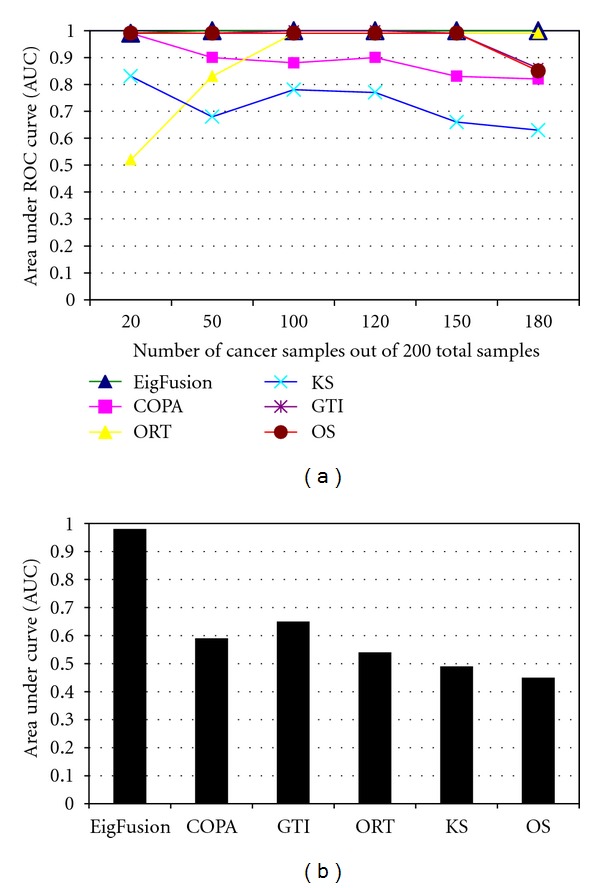
Evaluation of EigFusion performance on synthetic and real cancer data, AUC values are used to assess the performance of fusion gene detection methods. ROC curves were plotted as 1-specificity versus sensitivity of the methods. We plotted ROC curves for each method in several cancer samples size (*x*-axis) and found the area under the curve (AUC) as a measure of performance. (a) Using synthetic data, COPA and KS showed poor performance over all cases; on the other hand, ORT, GTI, and OS showed that poor performance is affected by the ratio of the size cancer samples to normal samples. (b) Applying all the methods on real prostate data (Singh data) showed that EigFusion outperforms the other methods.

We compared the performance of EigFusion, Kolmogorov-Smirnov (KS) statistics, OS, ORT, COPA, and GTI under different cancer sample sizes (Figure 2(a)). COPA was implemented as the 80th percentile of expression values after transformation of all data points using overall median and median absolute deviation for a given gene. We used 80th percentile as it is a medium value between the 90th and 70th percentile values that are most commonly used in COPA, plus it showed to give best results on synthetic data. The other methods were implemented as explained in [16]. As shown in Figure 2(a), EigFusion, GTI, and OS have high performance across all the variation in the cancer sample size. ORT performed poor when cancer samples size were smaller than normal samples size, but the performance was improved when cancer samples size is larger than normal samples size. KS showed the poorest performance as it is not designed for gene fusion extraction. This supports our hypothesis that traditional biomarker extraction methods are not suitable for outlier gene detection task. Previous works [13, 16] showed that traditional methods like *t*-test and significant analysis of microarray (SAM) are not suitable for the functional gene rearrangement detection problem; thus, our results are in agreement with pervious work.

**Figure 3:**
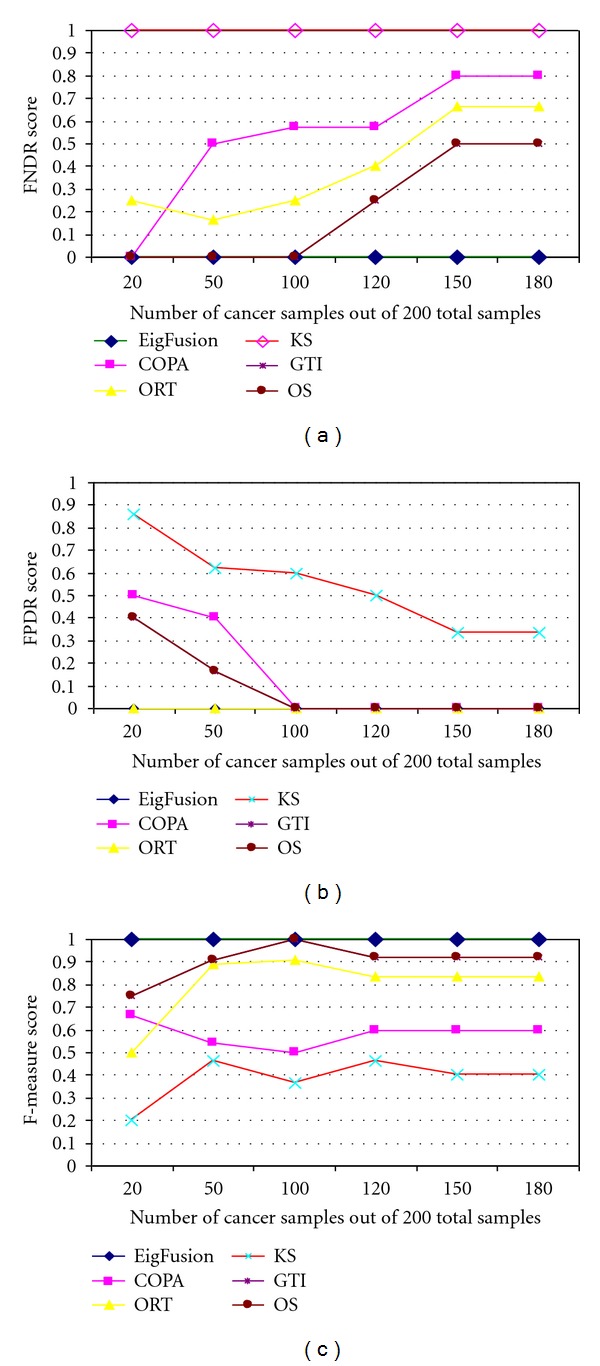
Evaluation of EigFusion performance on synthetic and real cancer data, AUC values are used to assess the performance of fusion gene detection methods. We used the (a) positive FDR (PFDR) and (b) negative FDR (NFDR) to assess FDR of each method under different cancer samples proportions. EigFusion showed to have zero FDR and f-measure value of 1. (c) We further assess the performance of the methods on real cancer data. We used Singh prostate cancer data with embedded test genes with different cancer proportions. We assessed the performance of each method based on their ability to identify test_10_ and test_20_ test genes.

EigFusion method showed to be very sensitive to the number of outlier samples; samples that harbour amplified genes or deletion. It can detect genes with outlier percentage of 1%, unlike the existing methods that require larger outlier percentage as they sum the values greater than IQR. When the cancer sample size was more than 50, most methods ranked *test*_2_, *test*_5_, and sometimes *test*_10_ at the bottom of the list. EigFusion showed to be very sensitive to such cases. We think this is very important as most of the gene fusion cases are very rare and occur in less than 5% of cancer patients [3]. Results in Figures 3(a) and 3(b) showed that EigFusion has zero false discovery rate compared with the other methods. Though we could not compare the performance of EigFusion to GTI and OS using AUC values as they showed very similar performance, f-measure (Figure 3(c)) showed a distinguished profile for EigFusion.

### 3.2. EigFusion Performance Evaluation on Prostate Cancer Data with Embedded Test Rearranged Genes

To test the models on real cancer expression data, we used gene expression data of 12600 probes in 59 prostate cancer samples and 87 normal samples [18] with embedded test genes. We used five test genes that have 10, 20, 30, 40 or 50 samples with rearranged genes. Good models should rank *test*_10_ and *test*_20_ high as they have small subset of samples with potential fusion. The other test genes have high expression in most of the samples and thus should not be ranked high as they would lead to false positive discovery. We showed that EigFusion outperformed the other methods on real data (Figure 2(b)) and it ranked *test*_20_ in the top of the list and *test*_10_ in fifth position. GTI ranked *test*_20_ in second position but was unable to rank *test*_10_ in the top 100. COPA also ranked *test*_20_ in the 20th position but was unable to rank *test*_10_ in the top 100. The other methods were unable to rank neither of the test genes in the top 100.

### 3.3. EigFusion Is Effective in Distinguishing between Biomarker Genes and Rearranged Genes

We compared the transformation function proposed in this work with COPA transformation function as they both transform all data points, unlike ORT, OS,  and GTI that only deal with values in the *O*_*i*_ set. We chose the case when cancer samples size is 50, as an example, and we compared the effect of the two methods on the *test*_50_ gene (Figure S1.C). In this case, *test*_50_ is a biomarker gene and should be ranked low. EigFusion standardizes the expression profile of genes based on the number of cancer samples, unlike COPA which standardizes the expression values based on the overall median regardless of the ratio between cancer and normal samples. EigFusion clearly shows how it can filter out biomarker genes.

### 3.4. Applications of EigFusion to Cancer Gene Expression Data

We next demonstrated the affectivity of EigFusion on real cancer data that harbour ERG rearrangement in around 50% of the samples. First SAM failed to detect ERG gene as outlier. EigFusion was applied on prostate cancer gene expression data as it is among the most heterogeneous types of cancer, both histologically and clinically. We used MSKCC Prostate Oncogenome Project data [19] which has 179 samples (131 primary, 19 metastatic, 29 normal). Our goal is to predict potential rearranged (amplified, deleted) genes that occur in primary cancer samples, and metastatic. To statistically assess the significance of the results, we randomly permutated the the sample labels for 100 time and then find a *P* value for each gene. Only genes with *P* value less than 0.001 we selected.

*TMPRSS2-ERG* gene fusions have been reported in approximately 50% of over 1500 clinically localized prostate cancer samples [3, 20]. This fusion replaces the 5' end of * ERG* with the 5' untranslated region of * TMPRSS2* which results in overexpression of * ERG* gene and downexpression of * TMPRSS2*. EigFusion is able to rediscover * ERG* fusion as the second top gene in the list (Figure S1.A). * SPINK1* is another gene that was predicted to be overexpressed, it plays a significant role in prostate cancer development. Tomlins et al. [21] first showed that high levels of serine peptidase inhibitor Kazal type 1 (*SPINK1*), which occurs in about 10% of patients with prostate cancer, were correlated with higher rate of cancer recurrence. We also found that * ERG* and * SPINK1* fusions have low cooccurrence rate (less than 2%) which agrees with the latest research findings about the role of * SPINK1* in * ERG*-negative rearranged prostate samples [21].

**Figure 4:**
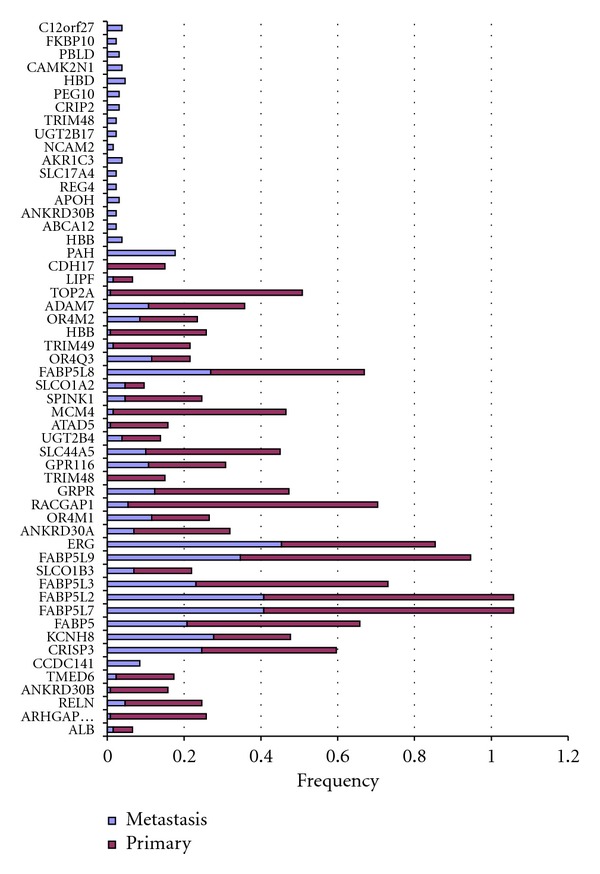
Genes altered in prostate samples, 54 genes are selected as overexpressed in subset of samples (primary or metastatic). Some genes showed to be overexpressed in only metastatic samples (genes with all red bars). Other genes showed to be overexpressed in both primary and metastatic but not normal samples (genes with red and blue bars). The frequency on the axis is the fractions of samples with rearrangement (overexpression in subset of samples) over all samples size. The red bars for example represents the frequency of gene rearrangement in primary samples.

We also identified other amplified genes which are potential candidates for rearrangements, such as * FABP5* (Figure S1.B) * KCNH8*. Many other genes such as * PAH*, *TOP2A*, and* CDH17* (Figure 4) showed to be amplified mainly in metastatic samples. We compared the set of rearranged genes from Taylor data with genes from Singh data [18]. We identified that * ERG, TFF3, FABP5, SPINK1, ISG15, *and* MRP4* as amplified genes representing potential rearrangements.

We further characterized gene rearrangements that are related to * ERG* fusion by grouping samples based on their ERG status: fusion-positive (ERG1) and fusion-negative (ERG0). We found several genes (*SPINK1, ETV1,PHA, *and* TFF3*) that are overexpressed only in subset of ERG0 samples. Also * FABP5* family showed to be more amplified in ERG0 samples, * KCNH8* and *GPR116* are more amplified in ERG1 samples (Figure S2). This is very essential to enable us to group prostate samples into subgroups: each with specific potential rearrangement signature, which may have prognostic implications. We also applied EigFusion on independent prostate cancer data [22] (455 samples) of known ERG fusion status. The samples were classified as 352 * ERG* fusion-negative prostate cancer samples (ERG0) and 103 * ERG* fusion positive prostate cancer samples (ERG1). We detected 18 potential oncogenic rearrangements associated with * ERG* negative samples. Interestingly, we found that * ETV1* is associated with * ERG0 *and has very low co-occurrence rate with ERG1. On the other hand, we found 25 potential gene rearrangements associated with * ERG* fusion positive. Interestingly, * SPINK5* was among the genes associated with * ERG *fusion.

We have also identified genes that are underexpressed in subset of the * ERG *fusion-positive samples. These genes could be the 5' partner of the gene fusions or could be deleted in the corresponding samples. We found also several genes underexpressed in subset of cancer samples like * KLK3, FOLH1, SPON2, A2M, *and *PCP4*. KLK3 has already been identified and used as a diagnostic biomarker. Thus, the other genes might be as important as KLK3. These genes could be either deleted in the corresponding samples or fused to one or many of the overexpressed genes.

We next asked if EigFusion can also be implemented to deal with other types of cancer. EigFusion was applied to ovarian cancer and leukemia gene expression data. We identified 94 putative rearranged genes in ovarian (Figure S3.A) and 88 genes in leukemia (Figure S3.B).

### 3.5. Rearranged Genes Are Functionally Associated

**Figure 5:**
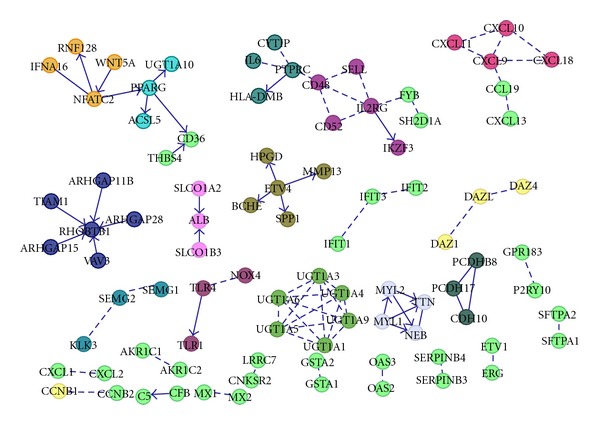
Integrating the discovered potential rearranged genes with functional protein interactions revealed functional modularity of the rearranged genes with enriched pathways.

 We next asked if the predicted rearranged genes are functionally associated and if they are enriched with particular biological processes. This is important to identify outlier genes that have influence on its neighbors. Thus, it helps to identify outliers that are influenced by rearranged genes. We integrated FPI network and the predicted set of potential rearranged genes to conduct core pathway analysis, based on the success of EigFusion in revealing common pathways alterations in prostate (Figure 5), ovarian (Figure S3.A), and leukemia (Figure S3.B) cancer. Results revealed that rearranged genes tend to form modules and share biological pathways. The tendency to form modules showed not to be random as we randomly selected 500 genes for 100 times and we did not observe any module enrichment in any of the 100 trials.  Table 1 describes that enriched pathways for the rearranged genes in the three cancers using EigFusion. We analyzed the pathways for all interacted genes and not individual modules. Wnt-signaling and cadherin signaling are commonly altered. Ovarian samples are altered in * KRAS* which is a member RAS/RAF cancer pathway. Another gene is * GNAZ* which is a member of the G protein complex that are involved as modulators or transducers in various transmembrane signaling systems. Leukemia samples are altered in integrin pathways and ERBb receptor family that are part of the epidermal growth factor (EGF) receptor family of receptor tyrosine kinases. Leukemia samples are also altered in RAS/RAF pathway at * RAC1* gene, a GTPase which belongs to the RAS superfamily of small GTP-binding proteins. We have also used the top 100 genes identified by COPA and GTI (supplementary file) and found that the two lists did not show any significant enrichment of biological pathways.

### 3.6. Identified Outlier Genes Are Associated with Perturbed Cancer Pathways

**Figure 6:**
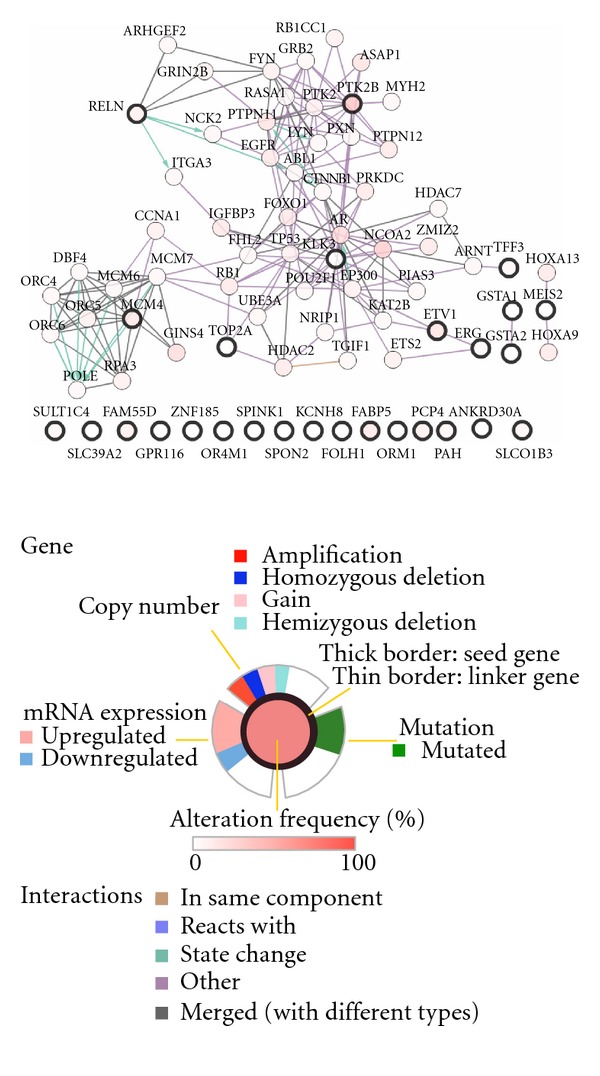
Functional modules of altered genes in prostate. Analyzing the rearranged genes by integrating functional protein networks and copy number alteration data revealed modularity of rearranged genes and high association with master regulators of well-known dysregulated pathways in cancer, such as AR, P53, and KLK3. Nodes with solid black border are identified by EigFusion.

 We next investigated if the predicted outlier genes are associated with master regulators that have been known to be altered in prostate and ovarian cancer. We integrated the copy number alteration (CNA) datasets to conduct pathway analysis of known altered cancer pathway. A search for altered subnetworks in functional protein networks identified several known pathways. Putative rearranged genes in prostate (Figure 6) showed to be highly associated with cancer master regulators * AR, KLK3, ERG, RB1, TP53, MCM4, FOXD1, PTK2B, NCOA2,* and* NCOA1* [23, 24]. Other genes like * FABP5, PCP4, SPON2, PAH, FOLH1, KCNH8, SPINK1, *and* GPR116* did not demonstrate any functionally associated modules, nor they are associated with master regulators. Genes rearranged in ovarian cancer (Figure S4.A) are highly linked to vital genes like * MYC, BRCA1, *and *PAX6* that were also altered in ovarian cancer. This provides further understanding of the deregulated pathways in cancer. We noticed that not all the identified rearranged genes are altered at the copy number level, but they are associated with altered genes. This indicates that these genes might not be altered but regulated by altered genes. We also conducted this analysis on the outlier genes identified by COPA and GTI. GTI genes showed to be associated with P53, MYC, RB1, and FYN. COPA genes are associated with TP53, MYC, RB1, and ACTA1. None of them showed any association with AR gene unlike EigFusion that showed that AR is most significant hub gene.

### 3.7. Validation of Outlier Genes Using Copy Number Variation Datasets

**Figure 7:**
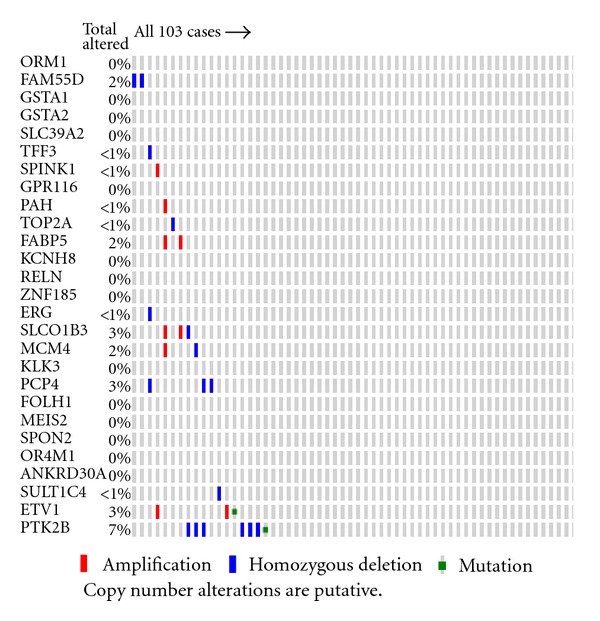
Validating prostate potential rearranged genes using prostate CNA revealed that half of genes are amplified or deleted in set of samples.

**Figure 8:**
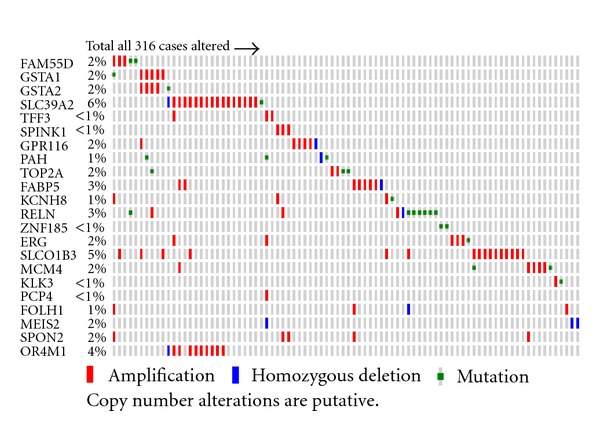
Validating prostate rearranged genes using ovarian CNA revealed that most of prostate rearranged genes are altered in larger portion of samples compared with prostate CNA.

After discovering rearranged genes in both prostate and ovarian cancer, we validated the genes using copy number alteration (CNA) datasets for the same sample set from which mRNA gene expression data was retrieved. We selected the top 27 (altered in more than 10% of samples) genes rearranged in prostate cancer and investigated the copy number alteration from CNA data (Figure 7). Approximately half (49%) of discovered genes were altered at the copy number level. We also observed that some of the genes were amplified and some were deleted. This shows that EigFusion can indeed identify amplified and deleted genes simultaneously. We then validated the prostate genes on ovarian CNA data. Interestingly, we found that most of prostate rearranged genes are also rearranged in ovarian cancer (Figure 8). We also found that the most significant genes identified by COPA and GTI have 19% and, 29% respectively, CNV. We then validated the ovarian rearranged genes using ovarian CNV and found that most of the discovered genes using EigFusion are copy number altered genes (Figure S4.B). Figure S4.B only shows the genes with the highest alteration rate. Ovarian genes did not show any significant alteration in prostate CNA data. Leukemia putative rearranged genes were not validated using CNA due to lack of CNA dataset of the same samples. 

### 3.8. Survival and Clinical Analysis of Patients with Putative Rearrangements

**Figure 9:**
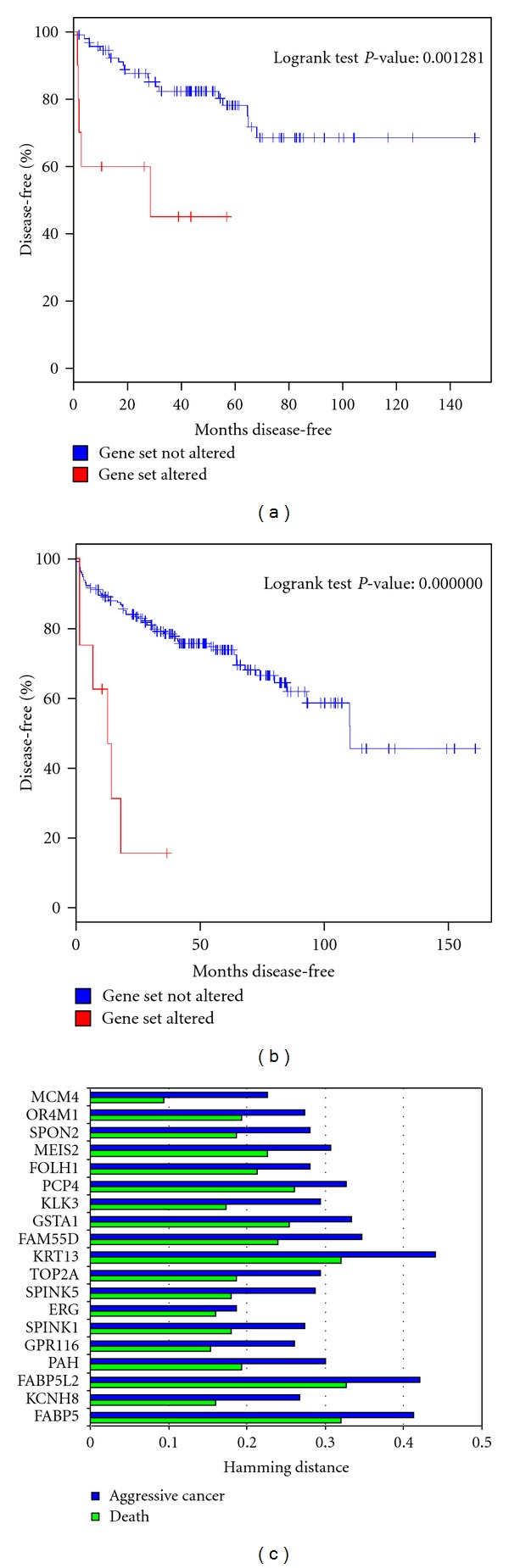
Clinical association of genes with death and aggressiveness of cancer. To understand the effect of alteration in gene expression, Kaplan-Meier survival curves are plotted to two sets of genes. (a) Is KM curves for all genes in figure top 25 genes altered. Samples with alterations demonstrated high risk disease. (b) Is KM curves using only ERG, SPINK1, KCNH8, and FABP5. Alterations in these four genes showed higher risk compared with the whole set of genes. (c) Hamming distance is used as a measure to find genes that have high association with death and aggressive cancer. Both death and aggressiveness were represented as vectors of samples. Distance shows how much gene's rearrangements vector differ from clinical vectors (death, aggressive). For example, ERG has distance of 0.16 to death vector; means that 84% of the samples of ERG fusion have death outcome.

 We further characterized the association of the predicted rearrangements and survival data (death versus no death) and clinical data (aggressive versus not aggressive). Aggressive samples are defined as samples with high Gleason score and are in cluster 5 as defined in Taylor et al. [19]. We represented genes with putative rearrangements as vector *V* defined over [0,1] of length *m*, where *m* is the number of samples. *V*(*i*) = 1 means that sample *i* includes a potential fusion. Death and aggressiveness were also represented as two vectors of length *m*. *V*(*i*) = 1 when the sample corresponds to death outcome or aggressive cancer. We found the hamming distance between the genes and the death and aggressive vectors to find how gene fusion is correlated with clinical outcome. We found that * ERG* is highly associated with death and aggressive cancer. 84% of samples with * ERG* fusion have death outcome and around 82% are aggressive samples. * MCM4* showed very significant association with death; 90% of samples with * MCM4* fusion have death outcome. * KCNH8, SPINK1, and GPR116* also have significant association with death (Figure 9).

**Figure 10:**
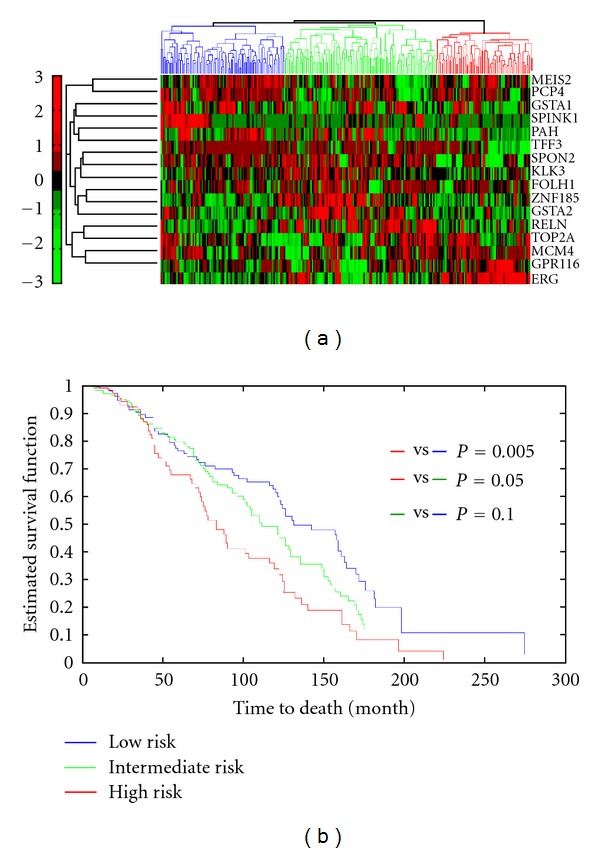
Hierarchical clustering of prostate rearranged genes. Validating the prostate rearranged genes on Swedish cohort revealed three prostate tumor subgroups with distinct rearrangement profile and different cancer specific death profiles.

Survival analysis (Figure 9) showed that samples with rearrangements in the identified 19 genes, that are altered in more than 10% of samples, showed to be at higher death risk than samples with no rearrangements (*P* = 0.00128, HR: 2.87). We reduced the gene set to * ERG, FABP5, KCNH8, SPINK1*, and we found that samples with rearrangements in these genes are even at higher risk of death (*P* < 0.0000001, HR: 4.12). ERG and SPINK1 are already associated with outcome; however, FABP5 and KCNH8 have not been associated with outcome. Here we showed that including FABP5 and KCNH8 as prognostic biomarkers improves aggressive cancer detection. Interestingly, rearrangements in prostate samples showed to be associated with survival in ovarian cancer. Ovarian cancer patients with rearrangements in prostate genes are at higher death risk (*P* = 0.03, HR: 1.4). We further used the Swedish prostate cohort data to assess if the rearranged genes are associated with cancer specific death. We only found the expression of 16 genes in the Swedish cohort. Clustering the gene expression data highlighted three distinct subgroups (low, intermediate, and high risk) (Figure 10). High risk patients are at higher risk for disease specific deaths compared to low risk patients (*P* = 0.005, HR : 1.89). No significant separation was observed between high and intermediate or low and intermediate groups.

## 4. Discussion

Here we argue that microarray gene expression data is a valuable source of information to discover outlier genes with potential functional gene rearrangements that have effect on the expression level of downstream genes. Since gene rearrangements are rare genetic translocation that affects a small sample of cancer patients and not all of them, it is feasible to discover genes that are overexpressed (amplified or fused) or underexpressed (deleted) in subset of cancer samples. Genes that are overexpressed in subset of samples are anticipated to be amplified or fused, and genes that are underexpressed in subset of samples are anticipated to be deleted. Unfortunately methods like SAM, *t*-test, *and so forth* that are developed to extract differentially expressed genes are not suitable to detect outlier genes. Previous works that aimed to identify gene rearrangements using bioinformatics approaches were limited to the identification of potential fused genes overexpressed in subset of samples and assessing the performance using synthetic data with embedded test genes. Herein, we followed the same approach by testing our EigFusion method on synthetic data with embedded tests. One might argue that real expression data does not follow certain distributions as in synthetic data. To address this point, we used real prostate cancer data with synthetic tests to test and compare methods. Unfortunately, there is no benchmark data that could be used in this study for performance evaluation purposes.

We compared the performance of EigFusion with all the methods in the literature that we are aware of that deal with outlier gene detection. One key factor that we considered and was not considered before is the size of cancer samples with respect to the size of normal samples. In this work, we showed that the ratio of cancer samples to normal samples significantly impacts the FDR. Existing methods such as COPA suffers from several drawbacks; first, the user defined *r*th percentile. Second, COPA is individual gene based method, and, most importantly, it fails to distinguish between biomarkers and genes with potential rearrangement especially when *S*2 is greater than or equal to *S*1. This is because the median will be biased toward normal samples. ORT, OS, and GTI also suffered from the same drawbacks. ORT showed to prefer high cancer proportion, unlike COPA that showed a decreasing performance as the cancer samples proportion increases (Figure 3). Based on Figure (3), ORT has zero FPR, but high FNR. This is because it is able to give a low rank to all genes that have high expression in all cancer samples, and has high FNR because it was unable to detect fusion genes when the cancer sample size increases. GTI and OS performed equally and they are the closest to EigFusion; however, GTI and OS are unable to discriminate between rearranged genes and biomarker genes when the cancer samples are less than normal samples (Figure 3), and they are unable to detect rearranged genes when cancer samples size increases (Figure 3). Both OS and GTI showed to have high FPR when cancer samples are less than 100, and high FNR when cancer samples are more than 100, they perform best when the samples are equally grouped into normal and cancer samples. They both showed not to be affected by the variation in the size of cancer samples. They ranked the same test genes in the same order regardless of the cancer samples size variation. EigFusion is a new method to detect rearranged genes that we proposed in this work which showed to have better performance compared with other existing methods. EigFusion is able to overcome one of the drawbacks of the other methods, which is distinguishing between rearranged genes and biomarkers genes. EigFusion identifies both overexpressed and underexpressed gene in the same run. Thus, we think EigFusion is more generic to be used to identify genetic rearrangements in general that result in gene expression change. We also stress on the impact of cancer samples size with respect to normal sample size, that should be considered in any gene rearrangement prediction problem.

In our study, we aimed to characterize outlier genes and their potential functional gene rearrangements in several tumor types: prostate, leukemia, and ovarian. We first focused on functional gene rearrangements in prostate cancer patients (primary and metastatic) compared with normal samples (Figure 4). We found that large portion of these gene rearrangements occur in metastatic samples; only * CCDC141* showed to be overexpressed in primary cancer. * FABP5* gene is overexpressed in both primary and metastatic cancer. * FABP5* is associated with psoriasis; it is a chronic immune-mediated disease that appears on the skin, breast cancer, and metastasis. Examination of the clinical implications of * FABP5* rearrangements revealed that samples with * FABP5* rearrangements are at higher risk of death (*P* value = 0.0000001) compared with ERG rearrangements (*P* value = 0.18). Furthermore, * FABP5* is overexpressed in samples that have * TARP* and * KLK3* underexpressed, which indicates that * FABP5* might be fused to * TARP *and* KLK3*. * TARP* gene is embedded within an intron of the T-cell receptor-gamma (TCRG) locus, which encodes an alternative T-cell receptor that is always coexpressed with T-cell receptor delta [25]. * TARP* was identified to be expressed in a prostate-specific form of TCRG mRNA in human prostate and demonstrated that it originated from epithelial cells [25]. This clearly shows that there is specific rearrangement or alternative splicing mechanism that leads toward aggressive cancer. Further characterizing * FABP5 *and* TARP*, they are rearranged in ERG0 samples, which means that these two genes could be used to define distinct group of prostate cancer. Several studies showed that * C-FABP or E-FABP* is a metastasis inducing gene overexpressed in human prostate carcinomas [26]. * KCNH8*, another significant gene identified in this work harbours a binding site for ELK-1 transcription factor, which is one of the ets- transcrption factors family to which * ERG* belongs, in its promoter. This might explain the association between KCNH8 and ERG.

One of the problems bioinformaticians face is validating the proposed computational algorithm. In this work, we validated the identified potential rearranged genes using CNA datasets for the same samples from which microarray gene expression data was conducted. Large portion of the genes were copy number altered, either amplified or deleted in both prostate and ovarian cancer. Validating prostate genes on CNA of ovarian data showed interesting result; altered genes in prostate are also altered in ovarian but not the opposite. We also found that ovarian samples have higher alteration rate than prostate samples. Most of the ovarian genes are altered in more than 8% of the ovarian cancer samples; however, prostate genes are only altered in around 2–4% of prostate samples. This reveals that ovarian cancer is more heterogenous than prostate cancer.

Several other findings have emerged from our analysis, largely based on the opportunity provided by integrated analysis of functional protein networks. Putative rearranged genes are functionally related and form modules that are enriched in biological pathways, mainly RAS/RAF and cadherin signaling pathways. A second finding is that integrating functional protein networks with CNA data provides insights on to the dysregulated pathways. EigFusion was able to identify elements (rearranged genes) in dysregulated pathway, but integrating CNA and functional networks gave more insights into the dysregulated pathways as other altered genes, that EigFusion was not able to retrieve, were identified. Thus, we believe that integrating EigFusion with functional protein networks and CNA data would reveal and give detailed insights into the dysregulated pathways. One of the findings we were able to retrieve using the integrative approach is the nuclear receptor coactivator * NCOA2* that was previously shown to alter AR pathway in primary prostate tumors providing mechanism for its potential role as a prostate cancer oncogene [19].

Survival analysis revealed that patients with rearrangements in the identified set of genes are at higher risk of cancer specific death. Using rearranged genes in prostate cancer helped to identify three subgroups with distinct outcome and different rearrangement profile. Using ovarian rearranged gene expression did not show significant prognostic value. Overall, these discoveries set the stage for approaches to the treatment of prostate, ovarian, and leukemia in which rearranged genes or network are detected and targeted with therapies selected to be effective against these specific aberrations.

## 5. Conclusion

Discovering cancer rearrangements can ameliorate the dysfunctional components in cancer cells. EigFusion successfully detected outlier genes with potential amplification or deletion genes (rearranged genes) in subset of cancer samples in both prostate and ovarian using gene expression data. EigFusion is the only method that is robust against variations in cancer sample size. Several genes like * ERG, FABP5, SPINK1, KCNH8,* and* PAH* are highly associated with outcome data. This set of genes could be used as prognostic biomarkers for prostate cancer. * ADIPOQ *and* LY6H* are discovered to be rearranged in 14% and 23% of ovarian samples, respectively. Using CNA to validate the rearranged genes demonstrated that ovarian cancer patients have higher rate of alterations per sample. Most ovarian cancer patients harbour multiple several genes altered. Integrating functional protein networks assisted to reveal the modularity of the rearranged genes. This ameliorates the functional dysfunctional genes as components rather than single genes. Genes with rearrangements helped to identify three prostate cancer subgroups with distinct outcome. Finally, gene expression data is a valuable and widely available source of information to discover gene with potential rearrangements.

## Supplementary Material

The supplementary material file contains detailed description of the biological data sets used in this work including gene expression and biological networks. It also includes detailed description of the existing methods for outlier gene detection. We covered most of the tools we are aware of that deals with this problem and compared our proposed tool (EigFusion) with all the described methods in this supplementary file (COPA, OS, ORT, GTI). The file also includes supplementary figures that we did not include in the main manuscript.

## Figures and Tables

**Table 1:** Pathway enrichment analysis for rearranged gene, *P* values are in (), FDR < 0.005.

Prostate	Ovarian	Leukemia
Drug metabolism (<0.0000)	Receptor-ligand complexes (<0.0000)	Integrin cell surface interaction (<0.0000)
Retinol metabolism (<0.0000)	Cadherin signaling pathway (<0.0000)	Focal adhesion (<0.0000)
Toll-like receptor signaling (<0.0000)	Regulation of B-cell development (0.0001)	ECM-receptor interaction (<0.0000)
Estrogen responsive protein (0.0003)	Wnt signaling (0.001)	Wnt signaling pathway (<0.0000)
Receptor-ligand complex (0.001)	Protein kinase (0.001)	Signaling by PDGF (<0.0000)
Signaling by Rho GTPases (0.004)	Signaling by FGFR (0.002)	Formation of platelet plug (<0.0000)
P53 signaling (0.005)	PPAR signaling (0.004)	Regulation of bone mineralization (<0.0000)
FOXA transcription (0.005)	Calcium signaling pathway (0.004)	Pathways in cancer (0.0001)
