# Supplementary material for: Detecting Cancer Outlier Genes with Potential Rearrangement Using Gene Expression Data and Biological Networks
Source: Adv Bioinformatics. 2012 Jun 28;2012:373506. doi: 10.1155/2012/373506 (PMC3394389; doi:10.1155/2012/373506)
Supplement: Supplementary file 1 — The supplementary material file contains detailed description of the biological data sets used in this work including gene expression and biological networks. It also includes detailed description of the existing methods for outlier gene detection. We covered most of the tools we are aware of that deals with this problem and compared our proposed tool (EigFusion) with all the described methods in this supplementary file (COPA, OS, ORT, GTI). The file also includes supplementary figures that we did not include in the main manuscript. [file 373506.f1.pdf]

Detecting Cancer Outlier Genes with Potential Rearrangement Using Gene Expression Data and Biological Network

Mohammed Alshalalfa, Tarek A. Bismar and Reda Alhajj

Supplementary Methods

Gene expression datasets:

Herein, we give a full description of the gene expression datasets we used in this work. We used three prostate cancer gene expression data, one leukemia and one ovarian cancer. For prostate cancer, we used data from MSKCC Prostate Oncogenome Project that is available at the Gene Expression Omnibus (GEO accession number : GSE21032). The data we used in this study contains expression level of 26443 genes and 179 samples (131 primary cancer, 19 metastatic, and 29 normal samples). The second prostate data is from the prostate Swedish prostate cohort (GSE16560) that contains expression of 6144 genes and 455 samples. The third prostate cancer data is from Singh et al [2]; it contains expression of 12600 genes across 59 prostate cancer samples and 87 normal samples. All data was normalized using quantile normalization using matlab function (quantilenorm()) then log transformed (base 2).

We used leukemia data from GSE425 with 23125 genes in 119 AML samples. The final data we used is ovarian cancer from The Cancer Genome Atlas Project. Data was downloaded from the TCGA website (http://www.cbioportal.org/public-portal). In this data the gene expression and miRNA expression of 489 samples have been analyzed . Detailed description of the data is available in [4]. We also used DNA copy number data from both the ovarian cancer and MSKCC Prostate Oncogenome Project to validate our results.

Existing Methods

Tomlins et al [1] proposed a method called Cancer Outlier Profile Analysis (COPA) to detect fusion genes using microarray gene expression data. The idea behind COPA is simple, cancer samples that have promoter to oncogene fusion resulted in high expression of the oncogene in the corresponding sample, and since such fusions are rare, only a subset of cancer samples harbour fusions, depending on the cancer type. So, the problem of detecting gene fusions is mapped into finding genes that are overexpressed in a subset of samples. Tomlins et al [1] ranked genes based on their 75th, 90th, and 95th percentile after centering the gene expression data by subtracting the median and dividing by median absolute deviation (MAD). MacDonald and Ghosh [5] added an additional criterion to rank genes with ties in the previous rank. They assessed the difference between the 75th percentile of the tumor and normal samples, and then computed the sum of these differences for each gene pair. The resulted value quantifies how different the outlier pairs are from their corresponding normal samples. Also, their work identifies pairs of genes that have large number of mutually exclusive outlier(cancer) samples, but few or no normal outliers. Other variations of COPA to improve its performance are outlier sum (OS) [6], outlier robust t-statistic (ORT)[7] and percentile analysis for differential gene expression (PADGE) [8]. OS only uses genes with expression values above certain cut-off IQR (Interquartile range) value in cancer

samples. ORT is not much different from OS, they only differ in the way they standardize gene expression values as detailed in the methods section. PADGE uses several percentile values and then take the maximum value. A recent method called Gene Tissue Index (GTI)[9]was proposed to consider the number of outlier samples that have expression value greater than a cut-off value (IQR). GTI deals with each tissue separately and then identifies genes with the largest difference in the GTI between cancer and normal tissues.

Cancer Outlier Profile Analysis (COPA)

The COPA [1] statistic is defined as the rth percentile of the disease samples' standardized expression values using *r* = 75; 90; or 95 as suggested by the authors. Each gene(i) expression value is standardized by subtracting the median(i) and divided by the median absolute deviation (mad(i))

$$X_{ij}^{\wedge} = \frac{X_{ij} - median_i}{mad_i}$$

$$mad_i = median(|X_{ij} - median(X_i)|)$$

After standardization, COPA ranks genes based on their r^{th} percentile of cancer samples S1 qr($X^\wedge_{ij}$: j ∈ S1). The COPA statistic can be formulated as:

$$qr(X_{ij}^{\wedge}: j \in S1) = \frac{qr\big(X_{ij}: j \in S1\big) - median\ i}{mad_i}$$

COPA is very similar to t-test statistics but replaces the average by median and standard deviation by mad.

Outlier sums (OS)

Outlier sums [6] was introduced to improve the rth percentile factor of COPA. OS uses only samples of values greater than a cut-off value q75 + IQR. Set of samples of values greater than the cut-off value for gene(i) is defined as O_i

$$O_i = \{j: j \in S1, X_{ij} > q75_i + IQR_i\}$$

where q75 is the 75th percentile and IQR is (q75-q25).
Considering only samples in Oi for each gene, OS standardizes the expression value of gene(i) by subtracting the median(i) and dividing the result by mad(i). The final score is the sum of the standardized values of each gene.

$$OSscore_i = \frac{\sum_{j \in Oi}(X_{ij} - median_i)}{mad_i}$$

Though OS overcomes the problem of the rth percentile, it is still single gene based method and unable to distinguish between biomarkers and rearranged genes when S2 is greater than S1.

Outlier Robust t-statistic (ORT)

The outlier robust t-statistic [7] is very similar to OS. It replaces the overall median by the median of normal samples. They also defined a new mad by subtracting the median of each group from the values in that group and then find the overall median.

$$ORT_i = \frac{\sum_{j \in Ri}(X_{ij} - median_i^{S2})}{median\{|X_{ij} - median_i^{S1}| i \in S1, |X_{ij} - median_i^{S2}| i \in S2\}}$$

Where R is the set of outliers disease samples for gene(i) defined by

$$R_i = \{j: j \in S1, x_{ij} > q75_i^{S1} + IQR_i^{S2}\}$$

Ri unlike Oi only focuses on normal samples. Again we think that this method is not proper to discriminate between biomarkers and gene fusion.

Gene Tissue Index (GTI)

The GTI algorithm [9] weights the proportion of outliers by a robust measure of how outlying the outliers are in a single group. A GTI value of gene(i) for each group (S) is defined as

$$GTI_i^S = \frac{t_i^S * (X_i^S - B_i)}{|S| * X_i^S}$$

where t^S_i is the number of samples with expression values above the cut-off in group S, |S| is the total number of samples in group S, X^S_i is the average expression of the samples above the cut-off for gene(i) in group S, and B_i is the standard statistical outlier cut-off for gene(i) (q75 + IQR). Then for each gene, $GTI_i = GTI^{S1}_i - GTI^{S1}_i$ is calculated

References:

1. Tomlins SA, Rhodes DR, Perner S, Dhanasekaran SM, Mehra R, Sun X, Varambally S, Cao X, Tchinda J, Kuefer R, Lee C, Montie JE, Shah RB, Pienta K, Rubin MA, Chinnaiyan AM: Recurrent fusion of TMPRSS2 and ETS transcription factor genes in prostate cancer. Science 2005, 310:644-648.

2. Singh D, Febbo PG, Ross K, Jackson DG, Manola J, Ladd C, Tamayo P, Renshaw AA, DAmico AV, Richie JP, Lander ES, Loda M, Kanto_ PW, Golub TR, Sellers WR: Gene expression correlates of clinical prostate cancer behavior. Cancer Cell 2002, 1:203-209.

3. Bullinger , L: Use of gene-expression profiling to identify prognostic subclasses in adult acute myeloid leukemia. N Engl J Med 2004, 350:1605-1616.

4. Cancer genomes atlas research network T: Integrated genomic analysis of ovarian carcinoma. Nature 2011, 474:609-615.

5. MacDonald JW, Ghosh D: COPA cancer outlier profile analysis. Bioinformatics 2006, 22:2950-2951.

6. Tibshirani R, Hastie T: Outlier sums for differential gene expression analysis. Biostatistics 2007, 8:2-8.

7. Baolin WU: Cancer outlier differential gene expression detection. Biostatistics 2007, 8:566{575.

8. Li L, Chaudhuri A, Chant J, Tang Z: PADGE analysis of heterogenous patterns of differential gene expression. Physiol Genomics 2007, 32:154-159.

9. Mpindi JP, Sara H, HaapaPaananen S, Kilpinen S, Pisto T, Busher E, Ojala K, Iljin K, Vainio P, Bjorkman M, Gupta S, Kohonen P, Nees M, Kallioniemi O: GTI A novel algorithm for identifying outlier gene expression pro_les from integrated microarray datasets. PLOS One 2011, 6:e17259.

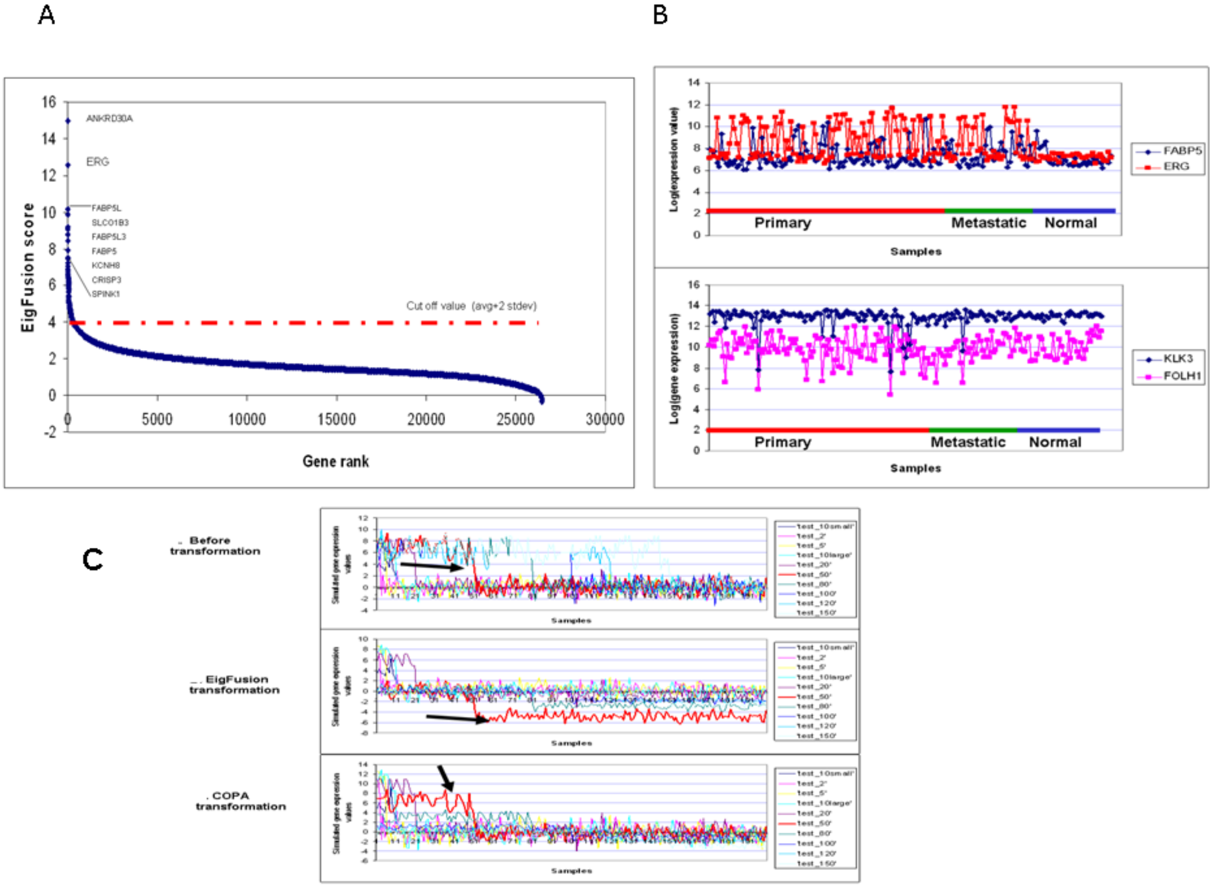

Figure S1: EigFusion gene ranking and transformation. (A)EigFusion score is plotted against the rank of each gene. The figure highlights the top genes that are predicted to be rearranged in Taylor prostate cancer dataset. We selected the cut-off value to be mean of all score values plus two times the standard deviation (avg+2stdev). (B)ERG and FABP5 are two examples of genes that are overexpressed in less than 50% of cancer samples (primary and metastatic). KLK3 and FOLH1 are two examples of genes underexpressed in less than 50% of cancer samples. (C) One of the challenges most methods face is filtering out false positive genes. We used $test_{50}$ gene to show how EigFusion is able to filter this gene out when the cancer sample size is 50. COPA transformation is unable to filter out$test_{50}$ gene due to the transformation function. This return to the importance of subtracting the median of cancer samples instead of the overall median.

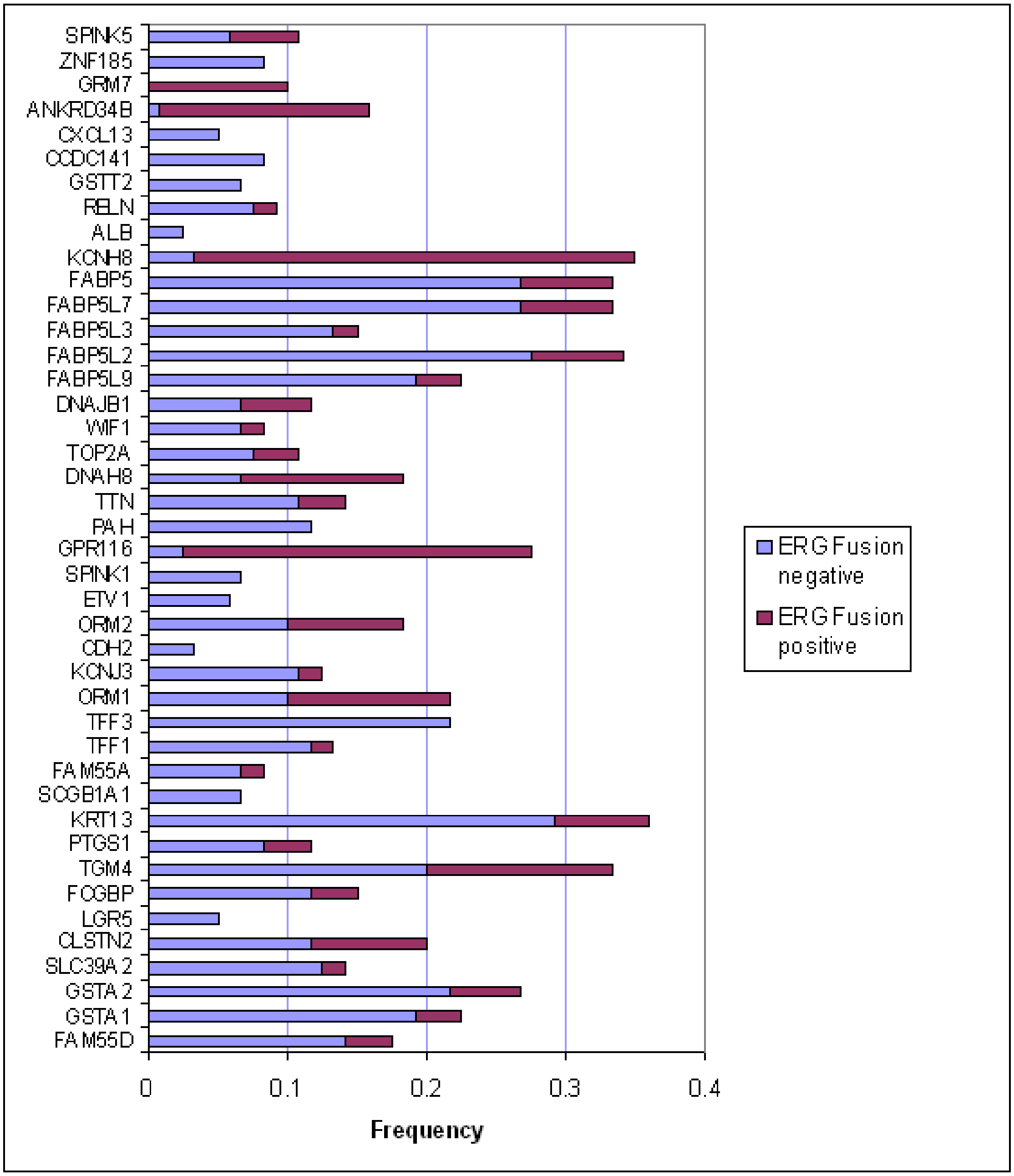

Figure S2: 43 genes are selected as overexpressed in ERG fusion positive or ERG negative fusion samples. Most genes are have higher frequency in ERG negative samples. KCNH8, GPR116 and ANKRD34D are more rearranged in ERG positive samples.

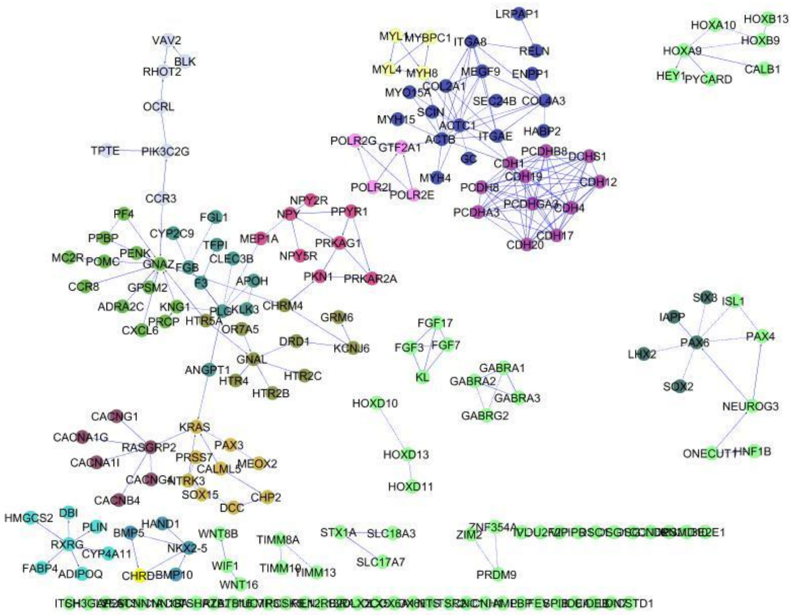

(A)

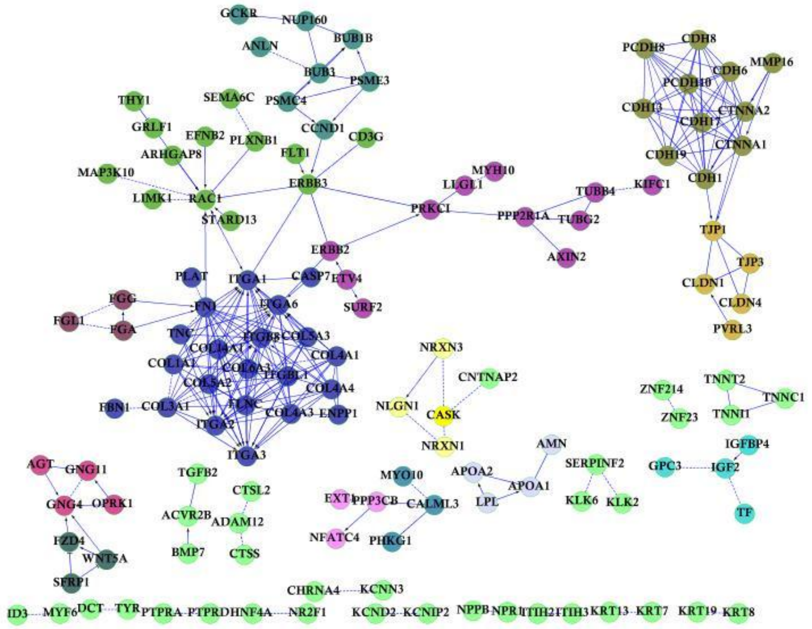

(B)

Figure S3: Functional altered gene modules in ovarian and leukemia. Integrating the discovered rearranged genes with functional protein interactions revealed functional modularity of the rearranged genes with enriched pathways in both ovarian (A)and leukemia tumor(B)

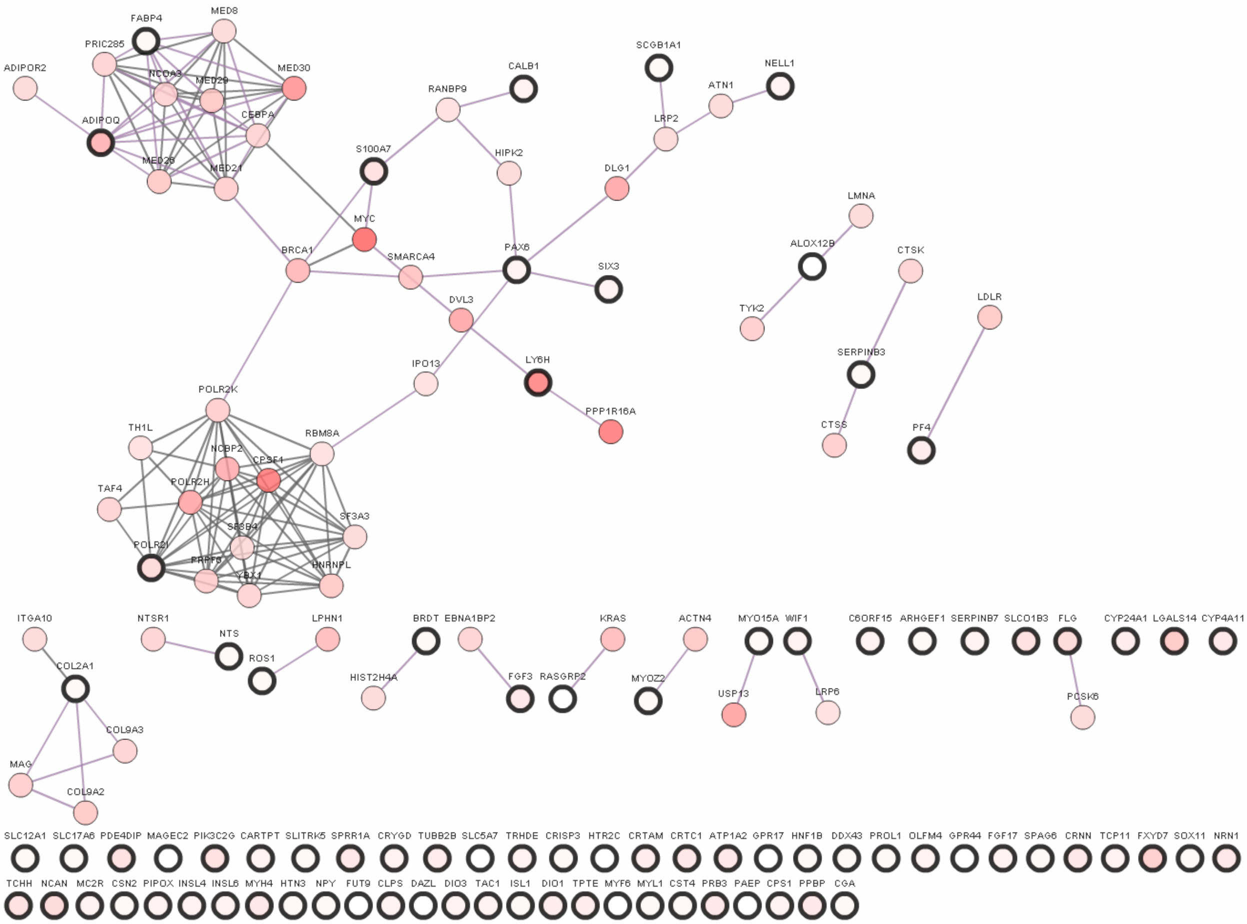
FABP4
MED8
PRIC285
MED30
ADIPOR2
NCOA3
MED29
ADIPOQ
CEBPA
MED26
MED21
SCGB1A1
CALB1
NELL1
RANBP9
ATN1
LRP2
S100A7
HIPK2
DLG1
LMNA
MYC
ALOX12B
CTSK
BRCA1
SMARCA4
PAX6
SIX3
TYK2
LDLR
DVL3
SERPINB3
IPO13
LY6H
POLR2K
PPP1R16A
CTSS
PF4
TH1L
RBM8A
NCBP2
CPSF1
TAF4
POLR2H
SF3A3
SF3B4
POLR2I
PRPF8
HNRNPL
YBX1
ITGA10
NTSR1
LPHN1
BRDT
EBNA1BP2
KRAS
ACTN4
MYO15A
WIF1
C6ORF15
ARHGEF1
SERPINB7
SLCO1B3
FLG
CYP24A1
LGALS14
CYP4A11
NTS
COL2A1
ROS1
HIST2H4A
FGF3
RASGRP2
MYOZ2
USP13
LRP6
PCSK6
COL9A3
MAG
COL9A2
SLC12A1
SLC17A6
PDE4DIP
MAGEC2
PIK3C2G
CARTPT
SLITRK5
SPRR1A
CRYGD
TUBB2B
SLC5A7
TRHDE
CRISP3
HTR2C
CRTAM
CRTC1
ATP1A2
GPR17
HNF1B
DDX43
PROL1
OLFM4
GPR44
FGF17
SPAG6
CRNN
TCP11
FXYD7
SOX11
NRN1
TCHH
NCAN
MC2R
CSN2
PIPOX
INSL4
INSL6
MYH4
HTN3
NPY
FUT9
CLPS
DAZL
DIO3
TAC1
ISL1
DIO1
TPTE
MYF6
MYL1
CST4
PRB3
PAEP
CPS1
PPBP
CGA
(A)

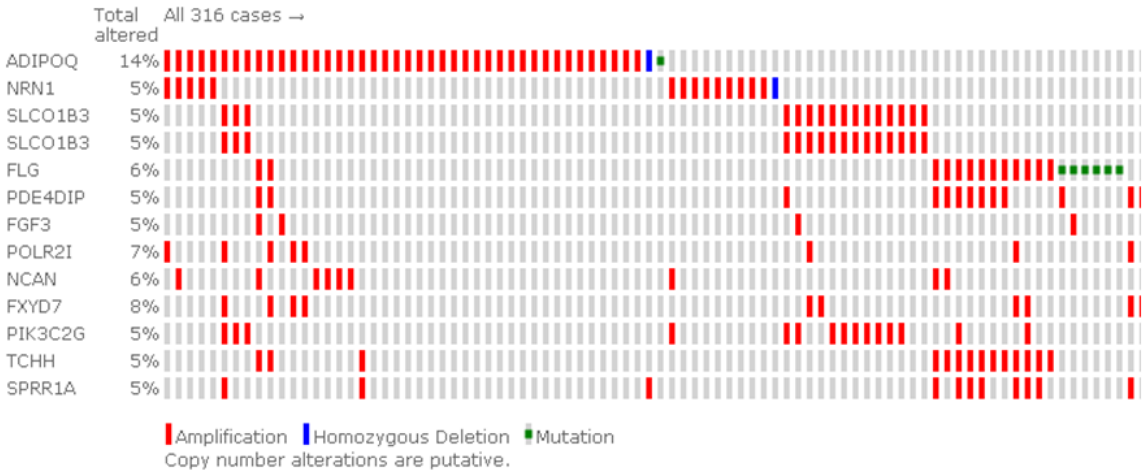

(B)

Figure S4. Functional association of altered genes in ovarian tumor to dysregulated pathways in cancer. (A)Integrating FPIs and CNA also revealed that ovarian rearranged genes forms modules that are linked with MYC and BRCA1. (B) Ovarian rearranged genes have high alteration rate compared with prostate.

Top outlier genes identified by COPA and GTI methods

COPA	GTI
ERG	ANKRD30A
CRISP3	ORM1
FABP5L2	LOC100133761
FABP5L9	SLCO1B3
LOC100128098	hCG_25653
FABP5L3	GPR116
CCDC141	SAA2
FABP5L7	LOC728027
FABP5	PPFIA2
PPFIA2	SPINK1
F5	PAH
LOC441416	TTN
TTN	LST-3TM12
LST-3TM12	ETV1
TFF3	SULT1C4
GRPR	DNAH8
PRC1	LOC441416
TDRD1	CCDC141
IFI6	ORM2
ANKRD30A	RELN
PAH	TOP2A
LOC100133678	LOC100133432
ATP5EP2	LRRC7
KCNH8	TTTY20
TOP2A	GRIN3A
PKIB	C12orf69
ATP8A2	CRISP3
LRRC9	HBB
PLA1A	CXCL13
ITPR3	AGR3
LOC100132184	LRRC9
LOC100132834	STAP1
LOC100133761	MYL2
CCNB1	TMED6
REPIN1	FAM55D
ARHGAP11B	ADAM7
AGTR1	MUC6
TGM4	ANLN
ADAMTSL1	TPX2
CHML	CYP4F8
C12orf69	SERPINB3
AMPD3	AKR1C3
SERPINB11	HBII-52-27

SLCO1B3	SNORD115-37
CENPI	LOC100132553
NFE2L3	ANKRD34B
HLA-DMB	SPP1
LOC100134041	EML6
TTTY20	CADPS
ORM2	SLC44A5
FABP5L10	ALB
OR51A7	TDO2
LOC100129489	DAZ1
FAP	ART4
CACNA1D	SAA1
LOC646851	LOC643069
RLN2	F5
NRN1	LOC728212
LOC730066	UGT1A1
CYTH3	FLJ45974
NELL2	IFIT1
LOX	DUX5
TRIP13	SERPINB4
LOC100129532	LOC100132029
LOC729885	ABCC11
LOC731228	LOC389740
LOC643069	SLCO1A2
ATXN2L	PROM1
LMNB1	UGT1A6
ACER3	SYCP2L
LOC647309	AK5
ARHGAP11A	GLYATL2
KHDRBS3	C12orf27
PCDHGB8P	LOC729384
TMEM178	LOC100131392
LOC100132769	SERPINI1
KLK12	CENPF
PCDHGA10	MS4A1
HIST1H3I	ABCA12
RCC2	SCGN
SPINK1	UGT2B17
ATP11A	PRLR
CST4	DAZ2
RNF157	OXGR1
LOC729960	LOC399939
RPL10L	LOC100134006
PRR11	LOC647309
CST2	UGT2B15
CARHSP1	LOC728160
LOC100128550	LOC728295

HOOK2	FABP5L9
LOC100134396	ANKRD30B
LOC100131731	TRIM48
COL28A1	HBA2
OAS3	HBA1
MS4A8B	MMP10
AK5	DAZ4
LOC100134769	DUX3
ADM	IFI44L
FABP5L8	SNORD115-7
TTK	RACGAP1
